# Olive-Derived Triterpenes Suppress SARS COV-2 Main Protease: A Promising Scaffold for Future Therapeutics

**DOI:** 10.3390/molecules26092654

**Published:** 2021-05-01

**Authors:** Hani A. Alhadrami, Ahmed M. Sayed, Ahmed M. Sharif, Esam I. Azhar, Mostafa E. Rateb

**Affiliations:** 1 Department of Medical Laboratory Technology, Faculty of Applied Medical Sciences, King Abdulaziz University, P.O. Box 80402, Jeddah 21589, Saudi Arabia; hanialhadrami@kau.edu.sa (H.A.A.); eazhar@kau.edu.sa (E.I.A.); 2 Special Infectious Agent Unit, King Fahd Medical Research Centre, King Abdulaziz University, P.O. Box 80402, Jeddah 21589, Saudi Arabia; hmsahmed@kau.edu.sa; 3 Molecular Diagnostic Lab, King Abdulaziz University Hospital, King Abdulaziz University, P.O. Box 80402, Jeddah 21589, Saudi Arabia; 4 Department of Pharmacognosy, Faculty of Pharmacy, Nahda University, Beni-Suef 62513, Egypt; Ahmed.mohamed.sayed@nub.edu.eg; 5 School of Computing, Engineering & Physical Sciences, University of the West of Scotland, Paisley PA1 2BE, UK

**Keywords:** triterpenes, olive leaves, SARS CoV-2, COVID-19, main protease, in-silico, molecular dynamic simulation

## Abstract

SARS CoV-2 pandemic is still considered a global health disaster, and newly emerged variants keep growing. A number of promising vaccines have been recently developed as a protective measure; however, cost-effective treatments are also of great importance to support this critical situation. Previously, betulinic acid has shown promising antiviral activity against SARS CoV via targeting its main protease. Herein, we investigated the inhibitory potential of this compound together with three other triterpene congeners (i.e., ursolic acid, maslinic acid, and betulin) derived from olive leaves against the viral main protease (M^pro^) of the currently widespread SARS CoV-2. Interestingly, betulinic, ursolic, and maslinic acids showed significant inhibitory activity (IC_50_ = 3.22–14.55 µM), while betulin was far less active (IC_50_ = 89.67 µM). A comprehensive in-silico analysis (i.e., ensemble docking, molecular dynamic simulation, and binding-free energy calculation) was then performed to describe the binding mode of these compounds with the enzyme catalytic active site and determine the main essential structural features required for their inhibitory activity. Results presented in this communication indicated that this class of compounds could be considered as a promising lead scaffold for developing cost-effective anti-SARS CoV-2 therapeutics.

## 1. Introduction

Severe acute respiratory syndrome caused by the enveloped virus coronavirus-2 (SARS CoV-2) is still a worldwide health disaster despite the recently introduced vaccines [1,2]. Hence, finding a cost-effective treatment is an urgent demand to support vaccines in controlling this rapidly evolving disease.

The viral main protease (M^pro^) is able to recognize and cleave specific amino acid sequences in the viral polyprotein replication complex (1ab). In addition, it is a highly conserved protein among coronaviruses [3], and thus it is considered an attractive target for the development of effective and specific therapeutics [4,5].

**Figure 1:**
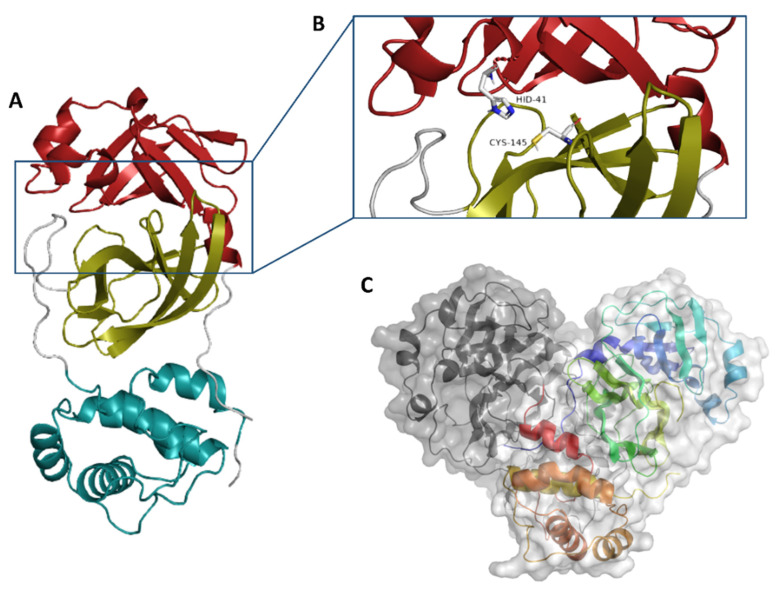
Monomeric structure of SARS CoV-2 M^pro^ (PDB code: 6LU7) showing its three main domains (I, II, and III; red, golden yellow, and cyan blue, respectively (**A**). M^pro^ active site showing the catalytic dyad (HID41-CYS145) (**B**). The dimeric active form of SARS CoV-2 M^pro^ (**C**).

Generally, M^pro^s of coronaviruses occur in a dimeric form (Figure 1C), and their catalytic activity significantly decreases if this conserved dimerization is inhibited by mutations [6,7]. Each individual monomer consists of three domains (domains I, II, and III), where the catalytic binding pocket occurs between domains I and II, while domain III is responsible for the enzyme dimerization [7,8] (Figure 1A,B). The enzyme active site contains a conserved HIS-41-CYS-145 catalytic dyad that is required for the protein cleavage. Hence, any modifications in these catalytic residues will eventually lead to a complete loss of the enzyme catalytic activity [9]. Several covalent inhibitors (i.e., covalent bond formation with CYS-145) have been developed [10], while non-covalent competitive inhibitors are much less investigated [11].

Being an essential pipeline for drug development, natural products have offered several anti-SARS CoV potential candidates that were able to target M^pro^. For example, betulinic acid, a plant-derived triterpene, exhibited promising inhibitory activity against the SARS CoV M^pro^ (IC_50_ 10 µM) [12]. Generally speaking, this class of natural products, in addition to plant-derived sterols, have shown good potential as antiviral agents [13].

Consequently, herein we investigated the efficacy of four olive-derived triterpenes, including betulinic acid against the M^pro^ of the currently widespread SARS CoV-2. Employing a number of in-silico experiments (docking and molecular dynamic simulation), we illustrated the main structural elements required for the M^pro^ inhibitory activity of this class of compounds, hence, offering a potential lead scaffold for further development and optimization to help in finding potential cost-effective therapeutics for COVID-19.

## 2. Results and Discussion

**Figure 2:**
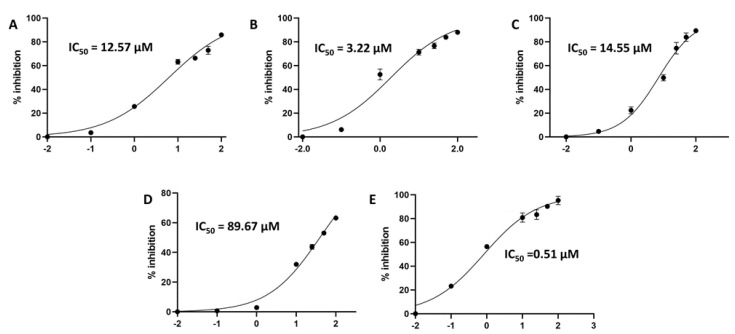
Inhibitory concentration 50 (IC_50_) of ursolic, maslinic, betulinic acids, betulin, and GC376 (i.e., reference M^Pro^ inhibitor) (**A**–**E**, respectively).

**Figure 3:**
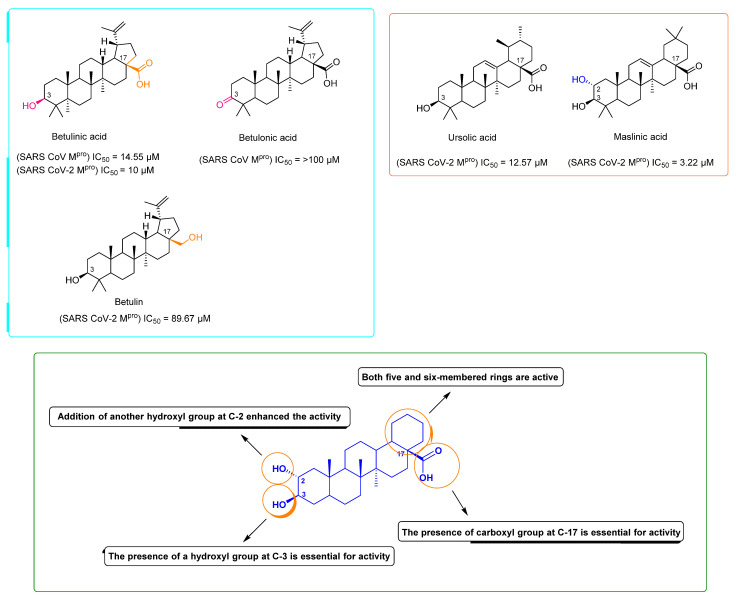
Structure of olive leaves-derived triterpenes investigated along with their IC_50_ against SARS CoV M^pro^ showing the main essential structural features required for the in vitro activity.

As a part of our continuous effort of finding natural products-based anti-SARS-CoV-2 therapeutics, we investigated four olive-derived triterpenes for their inhibitory activity against the viral M^pro^. Our rationale in the present communication was based on the previous findings of Wen and his co-workers [12], when they identified betulinic acid (lupan-type triterpene) as a potential SARS-CoV M^pro^ inhibitor (IC_50_ = 10 µM). Additionally, they highlighted that the presence of a hydroxyl group at C-3 as an H-bond donor is essential for the in vitro inhibitory activity (Figure 2). Hence, we tested betulinic acid together with three other olive-derived triterpene congeners (i.e., betulin, ursolic acid, and maslinic acid) against SARS CoV-2 M^pro^ to check if they are still promising scaffolds for the development of M^pro^ inhibitors against the newly emerged viral strain. Interestingly, we found that the lupine-type triterpene betulinic acid was still able to exert considerable inhibitory activity against the SARS CoV-2 M^pro^ (IC_50_ = 14.55 ± 1.3 µM), while betulin was far less active (IC_50_ = 89.67 ± 2.4 µM). These findings indicated that the C-17 carboxylate moiety is of particular importance for the inhibitory activity of this class of compounds (Figure 2 and Figure 3). Betulonic acid has been tested in a previous study on SARS CoV M^pro^ by Wen and his co-workers [12] and was found inactive. Although we did not test it against SARS CoV-2 M^pro^ in this study, we evaluated its binding mode and dynamic behavior just like the other tested congeners to extract the essential structural features required for the inhibitory activity of this class of compounds.

On the other hand, the tested ursane-type triterpenes, and ursolic and maslinic acids exhibited more promising inhibitory activities (IC_50_ = 12.57 ± 0.28 and 3.22 ± 0.26 µM, respectively) compared to the SARS CoV-2 M^pro^ positive standard GC376 (IC_50_ = 0.51 ± 0.08 µM). It was evident that the presence of an additional hydroxyl group at C-2 significantly increased the inhibitory activity of this type of compounds (Figure 2 and Figure 3).

**Figure 4:**
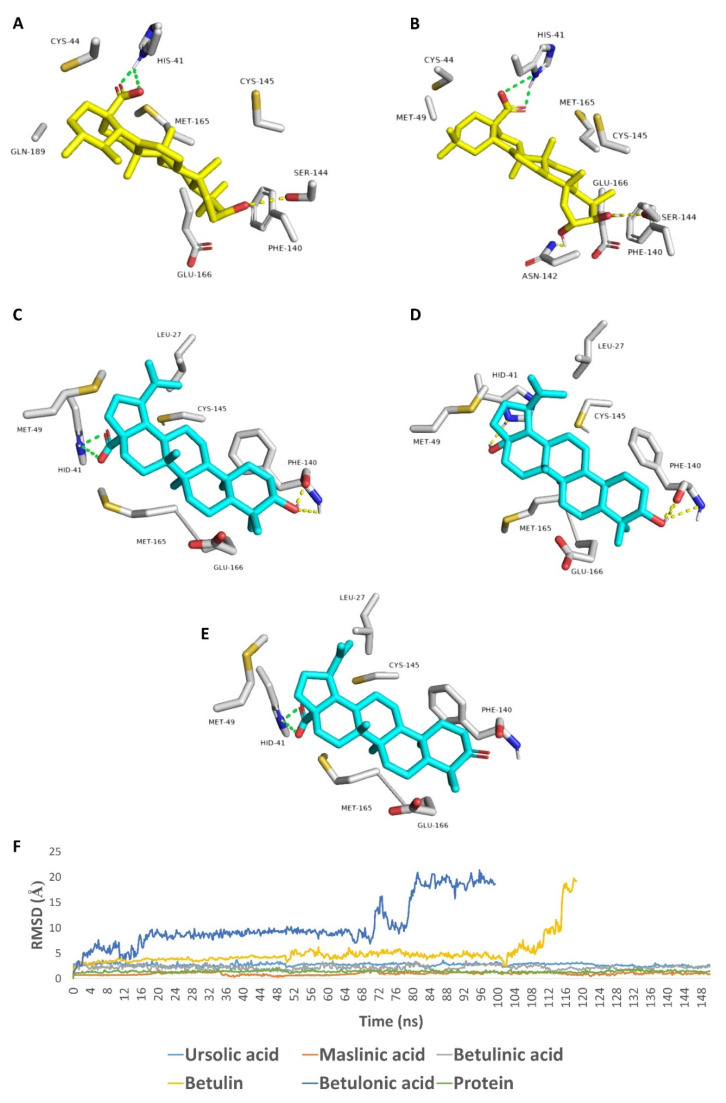
Binding modes of ursolic, maslinic, betulinic acids, betulin, and betulonic acid inside the M^pro^ active site (**A**–**E**, respectively). RMSDs of these compounds along with the apoprotein over 150 ns MDS (**F**).

To explain these in vitro results at a molecular level, we docked all of the tested compounds against the crystal structure of SARS CoV-2 M^pro^ (PDB: 6LU7) [10]. For this purpose, an ensemble docking protocol was used, taken into consideration the active site flexibility [8]. The top-ranking poses of each compound were convergent and represented a common ligand orientation (Orientation-A) (Figure 4) and were likely the most correct one as it was stable over a short run of molecular dynamic simulations (25 ns long). There was another orientation (Orientation-B, Figure S1A–D) of the docked compound represented by other lower-ranking poses. However, they were significantly less stable over the same period of MDS (Figure S1E).

As shown in Figure 3, which represents the top-ranking pose (Orientation-A), the C-17 carboxylate groups of betulonic, ursolic, and maslinic acids were able to form a salt bridge with HIS-41 (i.e., one of the M^pro^ catalytic dyad). In addition, the C-17 hydroxyl group of betulin was also involved in H-bonding with HIS-41. Concerning C-3-hydroxyl groups of the four compounds, they were involved as H-Bond donors with SER-144 in case of ursolic and maslinic acids, and as both H-bond donors and acceptors with PHE-140 main chain in the case of betulinic acid and betulin. If this C-3 hydroxyl group became oxidized and converted to a ketone (in the case of betulonic acid), it would no longer be able to contribute to any H-bondings, as the oxygen would lose its hydrogen and become oriented away from PHE-140 main chain. There were also a number of hydrophobic interactions between the compounds’ hydrocarbon bodies and LEU-27, MET-94, MET-165, and PHE-140, which supported the activity.

To further evaluate this binding orientation, we subjected those docking poses to longer MDS experiments (150 ns) and extracted the binding free energy (Δ*G*) of each compound (using the FEP method). As illustrated in Figure 3F, betulinic and ursolic acids were clustered together in the RMSD chart showing low conformational changes over the course of MDS (RMSDs ~ 2.42 Å). Maslinic acid showed lower conformational changes (RMSD ~ 0.95 Å), indicating that the second C-2 hydroxyl group further stabilized the whole molecule inside the enzyme’s active site. Trajectories’ analysis revealed that the interactions between C-17 carboxylate and HIS-41, and between C-3 hydroxyl group and either SER-144 or PHE-140 remained intact throughout the course of MDS (89% and 71% interaction time, respectively). Thus, these two types of interaction obviously played a significant role in making these compounds able to achieve stable binding with the M^pro’^s active site.

Regarding both betulin and betulonic acid, they showed significant fluctuations at the beginning of MDS till 70 ns, when betulonic acid started to leave the binding pocket from the C-3 side, and at 80.8 ns, the whole molecule became outside except for the carboxylate moiety. Similarly, betulin’s conformational changes were further increased until 110.4 ns, when the C-17 hydroxyl group was utterly detached and started to leave the binding pocket.

The calculated Δ*G*s of each compound reflected their corresponding stability inside the M^pro^ active site. Δ*G*s of betulinic and ursolic acids were convergent (−8.1 and −8.2 kcal/mol, respectively), while that of maslinic acid was lower (−9.3 kcal/mol). On the other hand, both betulin and betulonic acid became much higher Δ*G*s (−4.1 and −2.2 kcal/mol).

From the previous structural and dynamic information, it could be concluded that the presence of a C-17 carboxylate moiety together with ß- hydroxyl group at C-3 were essential structural features for this class of compounds to achieve a stable binding and, in turn, significant inhibition of M^pro^ activity (Figure 2 and Figure 3).

Recently, a number of other natural products have shown interesting inhibitory activity against SARS CoV-2 M^pro^ catalytic activity, particularly flavonoids [14,15,16]. For example, baicalein has exhibited potent micromolar inhibition (IC_50_ = 0.94 µM), and its crystal structure with M^pro^ indicated that its main molecular interactions were with GLY-143 and GLU-166 [15]. Surprisingly, this non-covalent competitive inhibitor was more potent than covalent peptidomimetics ones, such as molecule 5 h (IC_50_ = 4.2 µM) [17].

Olive leaves are a rich source of bioactive phenolics and triterpenes that have demonstrated a wide range of biological activities, including antiviral activities [18]. Previously, ursolic acid was reported to exhibit broad-spectrum antiviral potential [19,20]. Moreover, maslinic acid has shown in vitro inhibitory activity against HIV [21]. As discussed in the present study, triterpenoids, particularly those recovered from olive leaves, could be considered promising anti-SARS-CoV-2 leads that can suppress its M^pro^ catalytic activity, and we believe that the structural information presented here would support the development of potent and specific SARS CoV-2 M^pro^ inhibitors.

## 3. Materials and Methods

### 3.1. *Isolation of Compounds*

All triterpenes used in this study were isolated from the dried leaves of olive (i.e., *Olea europaea*) according to previously reported protocols [18,22]. Briefly, the dried leaves were washed thoroughly and then successively extracted with *n*-hexane:EtOAc (50:50). The produced extract was dried and then subjected to silica gel chromatographic column using CH_2_Cl_2_:MeOH (80:20) as a mobile phase to afford these four triterpenes. Structural characterization of these triterpenes was achieved using NMR spectrometry and comparison with authentic standards. To ensure the maximum purity during the in vitro testing, these triterpenes were purchased from Sigma Aldrich, St. Louis, Missouri, USA.

### 3.2. Ensemble Docking

AutoDock Vina docking software was employed in all docking experiments [23]. Triterpenes analogues were docked against the SARS CoV-2′s M^pro^ (PDB code: 6LU7) [10]. The enzyme’s active site was determined according to its co-crystalized ligand. To account for the active site’s flexibility, we used their MDS-derived conformers sampled every 10 ns for docking experiments (i.e., ensemble docking) [8]. Subsequently, we ranked top hits according to their average calculated binding energies. Docking poses were analyzed and visualized by Pymol software [23].

### 3.3. Molecular Dynamic Simulation

MDS experiments were carried out by Desmond v.2.2 [24,25] software that uses Maestro software [26] as a graphical user interface. Protein and protein–ligand systems were established by System Builder option, where they were enclosed inside an orthorhombic box of TIP3P waters together with 0.15 M of Na^+^ and Cl^-^ ions plus 20 Å solvent buffer. Afterwards, these prepared systems were energy minimized and equilibrated for 10 ns.

Desmond software automatically parameterizes inputted ligands during the system building step according to OPLS force field. For simulations carried out by NAMD [27], the parameters and topologies of the ligands were calculated either using Charmm27 force field by Ligand Reader & Modeler (http://www.charmm-gui.org/?doc=input/ligandrm, accessed on 15 February 2021) online software [28] or by the VMD plugin Force Field Toolkit (ffTK) [29]. Thereafter, the generated parameters and topology files were loaded to VMD so that it can readily read the protein-ligand complexes without errors and then conduct the simulation step.

Binding free energy calculations (Δ*G*) were performed via the free energy perturbation (FEP) technique. We first prepared all input files and script NAMD by the online-based CHARMM-GUI Free Energy Calculator (http://www.charmm-gui.org/input/fec, accessed on 15 February 2021) [28]. Afterwards, these inputs were loaded to NAMD for simulations, where the equilibration was achieved in NPT ensemble at 300 K and 1 atm (1.01325 bar) by Langevin piston pressure (for ″Complex″ and ″Ligand″) in the presence of TIP3P water model. For each compound, 10 ns FEP simulations were done (5 ns for equilibration + 5 ns for production) where the last 5 ns (i.e., the production step) of the free energy values were obtained for the final free energy values [28,29]. We did this simulation for the ligand alone, for the free protein alone, and for the complexed system (i.e., the enzyme bound to the ligand) to finally extract the Δ*G*. Finally, all the generated trajectories were visualized and analyzed by VMD software [29].

### 3.4. In Vitro Enzyme Assay

In vitro enzyme inhibition assays were carried out using SARS-CoV-2 main protease assay kit (Catalog #: 79955-1, BPS Bioscience, Inc., Allentown, PA, USA) and in accordance with the manufacturer’s protocol. GC376 was used as a reference standard inhibitor.

The produced fluorescence due to the protease cleavage of the substrate was observed at 460 and 360 nm for emission and excitation wavelength, respectively, using a Tecan Spark microplate reader (Switzerland). Briefly, 10 µL of test compounds of different concentrations were added into a 96-well plate followed by pipetting 30 µL of the diluted protease 15 µg/mL. The mixtures were incubated for 30 min at room temperature, and then 10 µL of the substrate was dissolved in the reaction buffer and added to reach a 50-µL final volume and 40-µM final concentration. The reaction mixture was then incubated for 4 h at 20 °C followed by measuring the produced fluorescence using a TECAN spark microplate-reading fluorimeter.

## 4. Conclusions

Plant-derived natural compounds remain a prolific source for discovering efficient drug candidates for a lot of serious health-related diseases. The COVID-19 pandemic is a serious issue that has been affecting the globe since the end of 2019. Even with few vaccines available, there is still a necessity for the discovery of promising antiviral agents. In this communication, we have highlighted the importance of plat-derived triterpenes as a promising structural motif for developing SARS-CoV-2 main protease inhibitors. Promisingly, maslinic acid exhibited almost half the inhibitory potential compared to the synthetic positive standard used for this screening (i.e., GC376, IC_50_ = 0.51 µM). We hope to screen a wider range of this structural class based on the structure–activity relationship pinpointed in this research to find safer and more potent inhibitor in the near future.

## Data Availability

Not applicable.

## Supplementary Materials

The following are available online, Orientation-B of binding of maslinic acid, ursolic acid, betulin, and betulinic acid inside the M^pro^ active site (**A**–**D**, respectively). RMSDs of these compounds along with the apoprotein over 25 ns MDS (**E**).

## Sample Availability

Samples of the compounds are available from the authors.
