# Supplementary material for: Olive-Derived Triterpenes Suppress SARS COV-2 Main Protease: A Promising Scaffold for Future Therapeutics
Source: Molecules. 2021 May 1;26(9):2654. doi: 10.3390/molecules26092654 (PMC8125615; doi:10.3390/molecules26092654)
Supplement: Supplementary file 1 [file molecules-26-02654-s001.zip › molecules-1190354-supplementary.pdf]

Supplementary Material

Olive-derived triterpenes suppress SARS CoV-2 main protease: A promising scaffold for future therapeutics

Hani A. Alhadrami,[1,2,†] Ahmed M. Sayed,[3,†] Ahmed M. Sharif,[4] Esam I. Azhar,[1,4] Mostafa E. Rateb[5,*]

[1] Department of Medical Laboratory Technology, Faculty of Applied Medical Sciences, King Abdulaziz University, P.O. BOX 80402 Jeddah 21589, Saudi Arabia

[2] Molecular Diagnostic Lab, King Abdulaziz University Hospital, King Abdulaziz University, P.O. BOX 80402 Jeddah 21589, Saudi Arabia; hanialhadrami@kau.edu.sa.

[3] Department of Pharmacognosy, Faculty of Pharmacy, Nahda University, Beni-Suef 62513, Egypt. Ahmed.mohamed.sayed@nub.edu.eg.

[4] Special Infectious Agent Unit, King Fahd Medical Research Centre, King Abdulaziz University, P. O. Box 80402, Jeddah 21589, SaudiArabia.

[5] School of Computing, Engineering & Physical Sciences, University of the West of Scotland, Paisley PA1 2BE, United Kingdom. Mostafa.Rateb@uws.ac.uk.

[†] These authors equally contributed to this work.
* Correspondence to MER (Mostafa.Rateb@uws.ac.uk

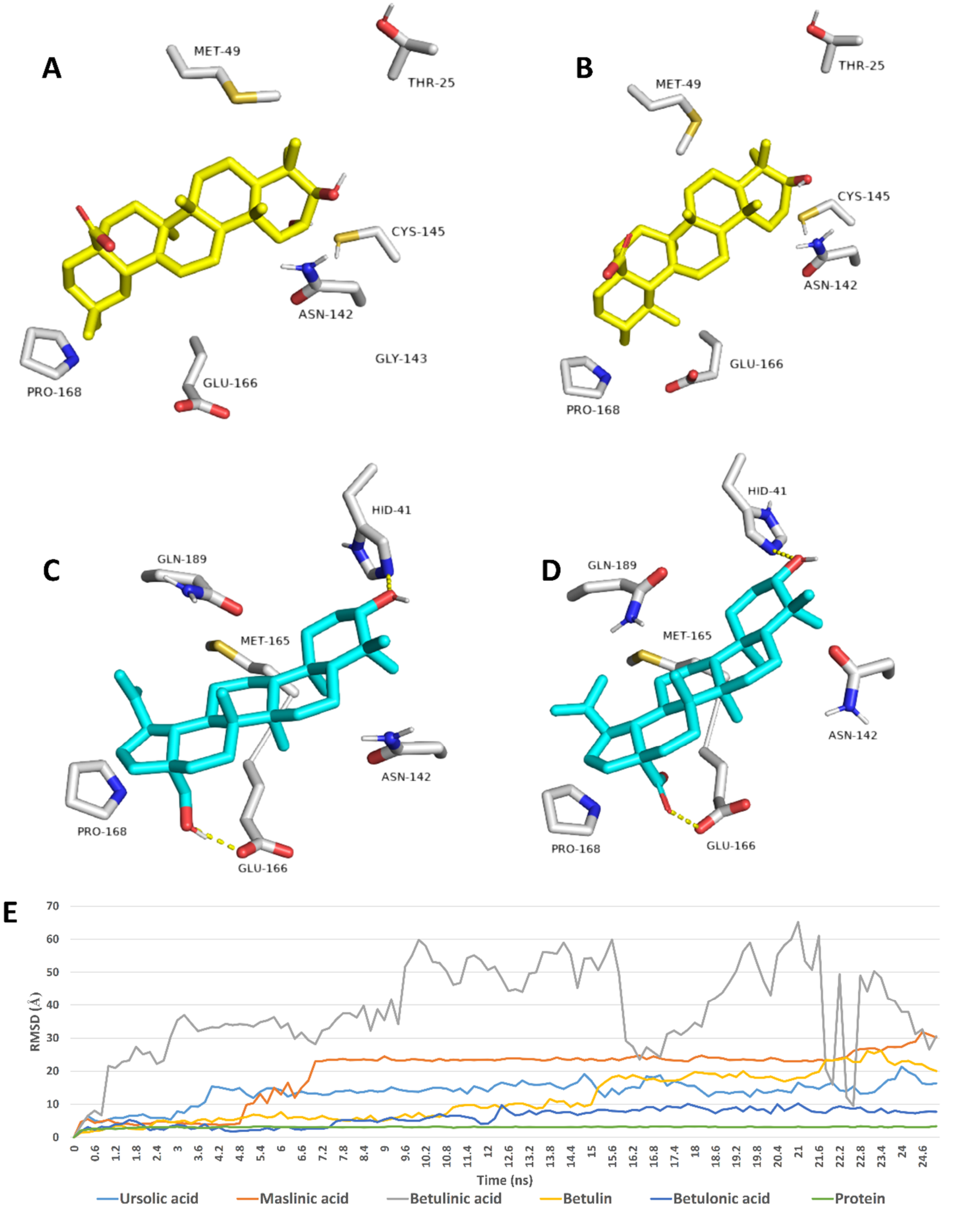

Figure S1. Orientation-B of binding of maslinic acid , ursolic acid, betulin, and betulinic acid inside the M^{pro} active site (A-D, respectively). RMSDs of these compounds along with the apoprotein over 25 ns MDS (E).
